# From Scale to Situated: Sociotechnical Imaginaries and the Configuration of Algorithmic Health Research

**DOI:** 10.1111/1467-9566.70205

**Published:** 2026-06-10

**Authors:** Kate Lyle, Gabrielle Samuel, Anneke Lucassen

**Affiliations:** ^1^ Nuffield Department of Medicine University of Oxford Oxford UK; ^2^ Global Health and Social Medicine Kings College London London UK

## Abstract

Contemporary healthcare systems generate vast volumes of data, with algorithmic interrogation promising disease prediction, improved diagnoses, and optimised treatment. Despite significant investment, biases in data used for algorithmic interrogation persist, leading to inequities in health outcomes. Scale alone cannot address these biases. Rather, considerations of the contextual dimensions of data need to be reflected upon. Nevertheless, calls for more data to ‘iron out’ such issues are common. Drawing on qualitative interviews with UK‐based health data researchers, we use Lucy Suchman's concept of configuration to explore how sociotechnical imaginaries of ‘big data’, which lead to calls for more data, are sustained, operationalised and enacted in everyday research practice. Specifically, we identify three interconnected processes that sustain these imaginaries: (1) risk‐oriented narratives that organise research around calculable futures; (2) decontextualising translation processes that align data with algorithmic requirements and (3) a persistent gap between algorithmic capacity and data availability. We conceptualise this third mechanism as a *productive* gap, as it continually renews commitments to scale by attributing limitations to insufficient data. We argue this gap represents a critical juncture for reconfiguration, revealing where assumptions about decontextualisation might be challenged to create space for more situated approaches to health data research.

## 1 Introduction

Contemporary healthcare systems have become sites of intensive data production, driven by great excitement about the new insights this data might unlock. Increasing volumes of data are being collected from expanding sources, including genomic sequencing, digitised health records, medical imaging, remote monitoring and wearable technologies, with the anticipation that they will transform understandings of health and disease (Subbiah 2023; Tran et al. 2019). Researchers deploy increasingly sophisticated computational approaches, including machine learning, natural language processing, and artificial intelligence techniques to analyse this data (Blasimme and Vayena 2020; Chen and See 2020; Loh 2018). These approaches are applied to aims ranging from predicting and preventing disease (Bengtsson et al. 2015), enabling more accurate diagnoses (Gurovich et al. 2019), modelling population health (Naghavi et al. 2010), optimising treatments and transforming drug discovery (Topol 2019) and supporting emerging practices such as digital phenotyping (Birk and Samuel 2020).

This turn toward data‐intensive health research is underpinned by a logic of scale, the assumption being that more data yields more robust, objective and actionable knowledge (Chalmers et al. 2016). Large national and global investments in biobanking and data infrastructure, such as UKBiobank, All of Us and the China Kadoorie Biobank, provide a necessary foundation for researchers to build massive datasets. Although challenges in working with large datasets are widely recognised, including representation gaps and algorithmic limitations, the prevailing assumption persists that sufficient data volume will ultimately ‘iron out’ these problems (Broussard 2023; Chalmers et al. 2016).

Yet, despite massive investments in data infrastructure and computational sophistication, systematic biases continue to be reproduced and amplified in health data research, through practices that shape both who is represented in datasets and how they are interpreted. Bias is embedded across multiple stages of data collection and algorithm development, including the curation of datasets that disproportionately draw on patients with greater access to healthcare, the use of standardised clinical categories that obscure important differences in how conditions present across patient groups, and the training of algorithms on such uneven data, leading to poorer performance for underrepresented groups (Norori et al. 2021). These dynamics are evident in existing research. For example, Flores et al. (2023) showed how public health surveillance tools replicate societal prejudices embedded in training data, whereas Boyd et al. (2023) demonstrated how structural bias in electronic health records reinforces inequities in care access and outcomes, particularly for marginalised populations. Algorithmic diagnostic systems, likewise, often exhibit differential accuracy across demographic groups (Cross et al. 2024). This illustrates that data are not neutral but are shaped by the conditions of their production (Gitelman 2013).

Lopez (2021) distinguished three forms of bias in data research: technical bias, arising from measurement errors or outdated data; socio‐technical bias, rooted in structural inequalities in how data is categorised or collected and societal bias, in which data reproduces wider social injustices. Such biases have cascading effects that extend far beyond academic concerns, undermining both scientific progress and equitable patient care. When research systematically excludes or misrepresents particular populations, the resulting knowledge base becomes inadequate for addressing their health needs and risks widening existing health inequalities (Epstein 2007; Krieger 2003). Efforts to address these problems often focus on technical interventions, such as refining datasets or recalibrating models, but these approaches are increasingly critiqued as insufficient. Broussard (2023), for instance, argues that technical ‘fixes’ frequently obscure the institutional and societal dynamics that produce bias in the first place. Without confronting these underlying structures, such fixes risk reinforcing rather than resolving inequity.

The persistent reproduction of bias and inequities in health data research is reflective of broader societal structures, and therefore scale alone cannot address the social contexts shaping health outcomes. These persistent structural biases raise important questions about how knowledge is produced, validated and used in data‐driven health research. Rather than pursuing ever‐larger datasets, we suggest these challenges call for more situated approaches that recognise health as embedded within the social, cultural, environmental and political contexts in which it unfolds. We use the term *situated* to emphasise that knowledge and data practices are not merely influenced by context but actively produced through specific social, material and institutional arrangements (Haraway 1988; Suchman 2007). The importance of this contextual understanding cannot be overstated; as McGilchrist (2019) reminds us, neglect of context is one of the greatest philosophical disasters of the modern age. While such perspectives are well established in health inequalities research (Nettleton 2021), we argue they must also extend to the ways health data itself is generated, interpreted and mobilised.

Situated approaches to quantitative data analysis and algorithmic research are gaining traction in other fields. In digital media studies, Rettberg (2020) proposes ‘situated data analysis’ as a framework for examining how the same data is constructed, framed and processed differently across multiple platform levels, revealing distinct power relationships as it moves from personal to aggregate uses. Similarly, in environmental modelling, Klein et al. (2024) advance situated modelling as an approach that challenges the notion of models as neutral tools, and instead treats them as situated knowledge practices shaped by the social and institutional contexts that determine what becomes visible and actionable. By contrast, health research remains largely committed to an objectivist understanding of data as discrete, complete and mobile entities that retain stable meaning across contexts.

This objectivist stance persists despite extensive social science research showing that data, science and technology are inherently situated and shaped by the contexts and practices of their production and interpretation—a point we take up in more detail below. The disconnect between this constructivist understanding of data and dominant health research paradigms suggests significant opportunities for collaboration. However, implementing such approaches is challenged by the pervasive influence of sociotechnical imaginaries that position ‘big data’ as an objective and universal solution to healthcare challenges (Henwood and Marent 2019). These imaginaries have implications for both research methodologies and the terms of interdisciplinary collaboration, and therefore understanding how they operate in everyday research practice is essential if alternative approaches are to be pursued.

Recent work has begun to illuminate how such imaginaries shape the broader organisation and expansion of data‐intensive healthcare. Drawing on Fiore‐Gartland and Neff's (2015) concept of data valences, Medina‐Perea et al. (2024) show how researchers' ideations of data interact with material investments in infrastructure and labour to drive the circulation and reuse of health data in the UK. By identifying these valences as drivers of expansion, they demonstrate how imaginaries of big data create favourable conditions for data flows between hospitals and universities. Building on this insight, this paper seeks to advance sociological understandings of how such imaginaries about data‐driven healthcare are enacted and reinforced through research practices.

Drawing on qualitative interviews with UK‐based researchers engaged in data‐intensive health research, and informed by Suchman’s (2012) concept of configuration, we analyse how these imaginaries are assembled through social, material and institutional practices organised around conceptualisations of risk that orient research toward prediction and future intervention. We then trace how sociotechnical imaginaries are enacted and stabilised within health data research, identifying three interconnected processes through which commitments to scale are continually renewed. In doing so, we illuminate junctures where reconfiguration may be possible and where space might be opened for more situated approaches. In what follows, we develop the conceptual framework for this study, drawing on work on sociotechnical imaginaries, risk and situated knowledge.

## 2 Sociotechnical Imaginaries and Situated Knowledge

Moving towards situated approaches to health data research represents a fundamental challenge that extends beyond methodological concerns. It requires a rethinking of what data are understood to be, and how knowledge claims are validated and mobilised. Yet contemporary health research is deeply shaped by powerful promissory discourses surrounding the transformative potential of ‘big data’ (Kitchin 2014). These promissories can be understood through the lens of sociotechnical imaginaries. Jasanoff and Kim (2015, 4) define sociotechnical imaginaries as ‘collectively held, institutionally stabilized and publicly performed visions of desirable futures, animated by shared understandings of forms of social life and social order attainable through, and supportive of, advances in science and technology’. In healthcare, these imaginaries coalesce around data‐driven solutions to complex health challenges, creating influential narratives about what legitimate research should look like and how scientific progress should unfold.

As Gardner (2023) demonstrates through his ethnographic study of hospitals, data‐driven imaginaries circulate across political, institutional and public discourse, positioning data as an objective solution to complex healthcare challenges. These imaginaries promise that comprehensive data collection and algorithmic analysis can deliver more efficient, effective and rational approaches to healthcare delivery and governance. Gardner highlights their ‘enchanting’ qualities that evoke excitement while appearing to align diverse stakeholder interests under a single technological vision. Importantly, these imaginaries gain persuasive power by providing strategies for demonstrating organisational legitimacy, positioning data as a powerful tool for policymakers and healthcare managers. Similarly, Elish and boyd (2018) argue that ‘Big Data’ and artificial intelligence operate through narratives that treat these technologies as transcending human limitations, creating what they term ‘magic’ that obscures the actual work of algorithmic analysis.

These imaginaries frame data as valuable not for what they currently explain, but for what they might reveal in the future. As Hoeyer (2019) argues, biomedical data are often accumulated speculatively: Their worth lies in the expectation that sufficient scale and technological advancement will eventually produce new and unforeseen insights. Importantly, this promissory orientation does not remain abstract, but is operationalised in practice through the concept of risk, which frames health outcomes as something that can be calculated, predicted and pre‐emptively managed (Armstrong 2023; Amoore 2016).

The turn towards risk as an organising principle in medicine is not new. Armstrong's (2023) historical analysis shows how population‐level risk probabilities have progressively reorganised clinical practice, shifting attention away from diagnosing and treating evident disease toward identifying and managing future possibilities. In this way, risk has become a legitimate object of medical knowledge and intervention in its own right, justifying action on the basis of statistical likelihood rather than lived experience or causal explanation, and reorienting clinical responsibility toward the anticipation and reduction of potential harms.

The growing prominence of algorithmic health research intensifies this turn toward risk through what Amoore (2016) conceptualises as ‘derivative life’. Amoore uses derivative life to describe a fundamental shift in how life becomes an object of governance and intervention. She argues that rather than acting on life as it is actually lived, with all its embodied complexity, social contexts and unpredictable trajectories, algorithmic systems operate on partial representations of life derived from digital data. These ‘derivatives’ are computational abstractions that claim to capture the essential actionable features of life while systematically stripping away the situated contexts in which life unfolds. Derivative governance operates through ‘anticipatory logics’ that alter both the site and timing of intervention. Rather than waiting for problems to manifest in lived experience, algorithmic approaches enable pre‐emptive intervention through computational derivatives like risk scores and predictive models that exist independently of their original contexts.

Amoore (2016) observes that this has driven a fundamental shift from ‘causation to correlation’; whereas previously research focused largely on understanding underlying mechanisms, algorithmic research often (although not always) prioritises risk through predictive performance, organising data around correlational relationships that enable pre‐emptive intervention. The focus on risk has important implications for knowledge production, ontologically and epistemologically positioning data as objective, discrete and uncontested, and framing health as something that can be abstracted into calculable correlations that travel across contexts without loss of meaning. These assumptions align closely with computational approaches that prioritise standardisation, abstraction and scalability. In this way, risk not only organises research practice but also shapes which ways of knowing health are legitimised and which are excluded.

Furthermore, extensive work in the field of science and technology studies (STS) has long demonstrated that even the most technical work is fundamentally relational, emerging from ongoing negotiations between diverse actors rather than from data speaking for itself (Knorr‐Cetina 1981; Latour 1987; Latour and Woolgar 1986; Pickering 1995; Traweek 1992). Feminist STS extends this work to show that all knowledge is situated: Researchers' positions in social and material relations fundamentally shape what becomes visible and knowable (Haraway 1988; Harding 1991). Most notably, the work of Donna Haraway and Lucy Suchman provide frameworks for understanding the situated nature of knowledge.

Haraway (1988) challenges the epistemological foundations of objectivity itself, arguing that claims to objective knowledge—what she calls ‘the view from nowhere’—represent particular historically located perspectives that conceal their own partiality. She terms this the ‘god trick’, a perspective that obscures the embodied partial nature of all knowledge claims while simultaneously privileging certain ways of knowing over others. Suchman (2007) extends this critique to human behaviour through her concept of ‘situated practice’, which reveals the fundamental misalignment between computational systems' predetermined logic and the contextual nature of human action. She distinguishes between ‘plans’, as the predetermined sequences that computational systems execute, and ‘situated actions’, as contextually responsive behaviours that characterise human activity. Context is not external noise to be controlled but the essential resource that makes action meaningful. Although feminist STS foregrounds the epistemic importance of context, computational approaches premised on the assumption that algorithmic analysis can transcend the limitations of data are often designed in ways that obscure or displace context. Understanding how such obscuring or displacement occurs is the central objective of this paper, and crucial if more situated forms of knowledge are to find purchase within research practice. To do this, we need to better understand how arrangements are assembled and sustained. Lucy Suchman's concept of *configuration* provides a way of doing so.

Suchman (2012) uses configuration to describe the socio‐material assemblages through which technologies come to hold together particular ways of knowing and acting, encompassing technical components, social meanings, institutional practices and future visions. Technologies do not gain significance from their technical properties alone but from the broader ideas people hold about what they will accomplish. At the same time, the material existence of technologies helps to stabilise and legitimise those very visions. This mutually reinforcing relationship means that imaginaries are not simply projected onto technologies but are actively sustained through their design, deployment and everyday use. To understand any technology, Suchman therefore directs attention to ‘the figure at the heart of a given configuration’. That is, the core ideas and orientations that organise how heterogeneous elements are brought together and held in place.

Importantly, configuration functions not only as an analytical lens but also as a way of identifying possibilities for intervention. As Suchman argues, examining configurations involves tracing ‘the relations that they hold in place and the labours that sustain them,’ thereby opening space for the ‘material–semiotic reconfigurations required for their transformation’ (Suchman 2007, 57). In the context of data‐driven health research, the pervasiveness of imaginaries that position data and algorithms as objective solutions creates significant barriers to approaches that foreground the partial, contextual and value‐laden nature of knowledge production. We argue that creating space for more situated approaches therefore requires examining how these imaginaries are embedded in everyday research practices and infrastructures. Making such configurations visible also opens the possibility of imagining how research might be organised otherwise—what Haraway (2016) terms ‘other worldings.’ This aligns with recent calls in STS to move beyond critique and engage more directly with how socio‐material arrangements are sustained in practice and how they might be reconfigured (Lyle 2020; Zuiderent‐Jerak 2015). Rather than treating abstraction or decontextualisation as inevitable features of algorithmic research, this paper examines how they are actively produced through specific sociomaterial configurations. Drawing on qualitative interviews with health data researchers, we use the concept of configuration to trace how these arrangements take shape in practice, and to identify where possibilities for alternative more situated approaches might emerge.

## 3 Methodology

### 3.1 Research Design

This study draws on qualitative interviews with UK researchers engaged in data‐intensive health research. The study was originally designed to explore environmental sustainability, but analysis of the interviews revealed the central role of sociotechnical imaginaries in shaping and being reinforced by researchers' everyday practices. To examine this emergent finding, we captured participants' perspectives on their data‐intensive work and the future‐oriented visions that guide and inform it. The environmental sustainability aspects of the study are reported elsewhere (Samuel 2023, 2024).

### 3.2 Participant Recruitment

Participants were recruited through purposive sampling, targeting UK‐based researchers involved in health‐related research using data‐intensive approaches. Participants were identified via several routes, including: a publicly accessible list of successful applications to access the UKBiobank resource; a publicly accessible list of individuals involved in the Genomics England clinical implementation partnerships; various bioinformatics journals, including, for example, Biodata and Mining and the Journal of Biomedical Informatics; Web of Science searches using keywords associated with biosensing research (‘mobile sensing’; (‘wearables’ and ‘health’); (‘biosensors’ and ‘health and data’); (‘digital phenotyping’); web searches for data‐driven health initiatives at various UK public and private institutions and organisations and snowballing. Through this comprehensive approach we identified 145 relevant UK‐based researchers and research consortia, all of whom were invited to participate via email. Twenty‐six researchers agreed to participate and completed interviews.

### 3.3 Data Collection

Semi‐structured interviews were conducted between January and March 2022, delivered either online or via telephone. All interviews except one were digitally audio‐recorded; the remaining interview was completed in written format at the participant's request. Interview duration ranged from 25 to 65 min, with the majority (*n* = 18) exceeding 40 min. The interview schedule explored several key areas: participants' professional backgrounds, their use of data‐intensive methodologies, the types and quantities of data employed in their research, data management practices (including access, collection, storage and processing) and the expectations, barriers and challenges associated with their data‐intensive research.

Whereas the field of algorithmic health research continues to evolve rapidly, including significant developments since our data were collected in early 2022, the analysis presented here focuses on broader sociotechnical configurations and imaginaries rather than on specific tools or platforms. The persistence of promissory narratives, risk‐oriented research logics and commitments to scale suggests that the dynamics we identify are not confined to a particular technological moment.

### 3.4 Participant Characteristics

The sample comprised 26 researchers representing diverse backgrounds within data‐driven health research. The majority were male (*n* = 21), reflecting the documented gender imbalance in this field. Participants represented various career stages, including nine professors, 13 research associates/fellows/lecturers/senior lecturers, one PhD student, one health research data manager and two researchers from small‐to‐medium enterprises (SMEs).

Participants were affiliated with 14 different universities and companies and represented a range of disciplinary backgrounds: clinical research (*n* = 6), engineering including artificial intelligence (*n* = 6), public health and epidemiology (*n* = 6), data science and bioinformatics (*n* = 4), health services research (*n* = 2) and data management and curation (*n* = 2).

### 3.5 Data Analysis

Interview recordings were transcribed verbatim and analysed using NVivo qualitative data analysis software. The analytical approach combined both inductive and deductive coding within an abductive framework (Timmermans and Tavory 2012). The first phase employed open coding to construct themes inductively from the data. This involved memo‐making and systematic scanning of interview transcripts to identify patterns in participants' experiences, practices and perspectives without imposing predetermined theoretical categories. The second phase applied deductive coding informed by the theoretical framework described above. This iterative approach enabled the development and refinement of themes while ensuring that emerging patterns were grounded in participants' experiences and theoretically informed interpretations were empirically substantiated. The integration of inductive and deductive approaches allowed for the identification of novel insights while maintaining theoretical coherence in understanding how sociotechnical imaginaries are sustained through everyday research practices.

## 4 Configurating Research Practices

Through participants' accounts of their research work, we explore how sociotechnical imaginaries about data‐driven healthcare are enacted and reinforced in everyday practice. We identify three interconnected processes through which these imaginaries are pursued and sustained, operating at different analytic levels. First, we explore how these imaginaries are operationalised through decontextualised narratives of risk which frame research aims and priorities in terms of calculable and predictable health outcomes and pre‐emptive intervention. Second, we show how this framing is enacted materially through processes of translation that strip away contextual information in order to render data compatible with algorithmic analysis. Third, a persistent gap between algorithmic capacity and data availability functions to sustain the imaginary by continually justifying further data collection, infrastructural expansion and ongoing commitment to data‐intensive approaches. Together, these processes illustrate how sociotechnical imaginaries are not simply articulated as visions of the future but actively worked through and reproduced in everyday research practice.

### 4.1 Narratives of Risk

Sociotechnical imaginaries operate through assemblages of diverse actors and technologies. To understand how these assemblages cohere, Suchman (2012) directs us to examine ‘the figure at the heart of a given configuration’, that is the central organising principle that gives meaning to diverse elements and practices. Below we show how narratives of risk are a persistent figure in participants' accounts, shaping how they mobilise data and its role in technological configurations. It is ‘risk’ and its potential to be managed that brings the data into meaningful existence.

Participants consistently emphasised data's potential to transform medical understanding and practice by revealing risk factors for disease. As one participant explained, these approaches are already operational within clinical practice:

In the prediction of cardiovascular disease or diabetes, I think the NHS has this algorithm already available. You can even access them from the website. You put your age, sex, do you smoke etcetera, and they can give you a scale of your risk. Then GPs, hospitals, rely on this risk to provide intervention or prevention programmes or certain measures for the high risk individuals.

(Interviewee 12)

The rhetoric of risk was reiterated across participants' accounts, bringing meaning to their data work by connecting it to potential interventions for managing risk (even if these interventions were aspirational rather than presently achievable). Sometimes this focuses on elucidating risks around treatment response:

[My research is] trying to see whether there are certain characteristics of patients that can predict when to treat them in hospital for atrial fibrillation…. When they treat them using what is called catheter ablation,… there are certain patients [for whom] it [works] forever. But there are other patients… [in whom] it occurs again, so they have to come back. So [we are trying to] develop models that are able to predict based on the characteristics of the patients, also based on the characteristics of the heart of the patient… whether a patient will go and come back or will never come back.

(Interviewee 4)

In other instances, the value in examining and elucidating risk comes from enabling treatment *before* the physical manifestation of disease:

I'm working on a project that uses some more advanced prediction methods to predict the risk of individuals developing eating disorder symptoms… So some characteristics at early adolescence could predict the development of binge eating, purging, vomiting, taking laxatives etcetera, over‐exercising … even before the onset of the symptoms.

(Interviewee 11)

In other cases still, the risk rhetoric focuses on optimising therapeutic selection and outcomes:

If someone comes into clinic we see them, we do a scan and we're able to say, well, this treatment is going to work for you, or this is what you can expect in the future. And we're absolutely convinced, we are absolutely sure this is the correct diagnosis, and this is the correct treatment. And then the patient goes away and gets better. That will be the ideal.

(Interviewee 3)

This focus on risk exemplifies what Suchman describes as the embodiment of rhetorical categories. By assembling diverse sociomaterial practices, including data, samples, algorithms, biological processes and clinical outcomes, around the concept of risk, these diverse human and non‐human actors make risk appear to be a natural category that exists independently in the world. Through this process, risk becomes reified as an organising logic that seems to naturally hold these elements together, obscuring that risk is a relational concept socially constructed to manage uncertainty. Importantly, this reification reconfigures the fundamental target of medical intervention. Rather than treating the physical manifestation of disease, risk itself becomes the primary object of medical action. This means that rather than the complex social and biological contexts where disease actually emerges, data‐driven approaches focus on technical data integration capabilities rather than domain‐specific knowledge. For example:

We are not experts in any of the clinical phenotypes, but we are really experts in integrating different multi‐dimensional data. And then apply machine learning artificial intelligence to come up with other predictors, or new models that give us insight into the aetiology of the phenotype.

(Interviewee 1)

Understanding this reification and focus on risk is important for explaining why situated approaches struggle to gain traction in health data research. When risk appears as a natural category requiring algorithmic calculation, alternative ways of organising health knowledge, such as approaches that foreground social context and situated understanding, appear unscientific or inadequate by comparison. The material assemblage around risk makes this particular way of understanding health seem inevitable rather than constructed, rendering invisible the social processes through which risk categories themselves are created and the contextual factors they systematically exclude.

Having explored how risk operates as the organising principle of health data research, we now turn to examine how researchers use algorithms to implement this vision in practice.

### 4.2 Processes of Translation

Building on this risk‐oriented framing, this section examines the practical and material work required to make data compatible with algorithmic analysis. The patterns and associations that researchers seek within the data can only be constructed through algorithmic processing designed to reveal relationships imperceptible to human analysis. As one participant explained, this computational mediation is essential for extracting meaningful information:

So you do imaging and then you extract features [data] and then you try to learn patterns from these features to answer a relevant question… these images, they are multi‐dimensional, and actually, there's nothing that you can see just by eyeballing. You have to pass them through a number of computational steps and models to extract the relevant information.

(Interviewee 10)

This process involves transforming data into mathematical relationships, as another participant explained:

In machine learning typically, you always have, so I often use the term model here, you could say a model is somewhat an algorithm. But it's essentially a statistical model… I can try to predict Y from X and that's what we're always doing. And a very simple model is just one variable, one parameter like weight that can be tweaked. …And the modern machine learning is usually you have lots and lots of different weights, lots of input variables that you don't sometimes even know what they are …And then I put a model on top of that, it's like a nest of models, there's lots of models in it.

(Interviewee 2)

The value of data, then, is tied to the unknown risk factors for disease that can be constructed through these algorithmic processes. To be able to organise data into patterns of mathematical relationship, algorithms require data in specific formats—data cannot be directly inputted into these systems in its original form. Participants described laborious processes of ‘translation’ (Callon 1986) through which they made datasets compatible with the computational tools used to interrogate them:

Making data good, you spend 95 percent of your time for that project on data, just processing it. Because the better it is, the better you are for the material learning. Because [running the algorithm] that's a very simple task. The most important task is getting the data in correct fashion.

(Interviewee 4)

This process of getting data into the ‘correct fashion’ represents systematic decontextualisation. Data must be stripped of its contextual particularities, generated through specific clinical encounters, particular institutional practices and local contexts and transformed into the standardised ‘input variables’ that machine learning algorithms require. What emerges is data divorced from the circumstances of its production, transformed into the abstract mathematical entities that can function across different computational contexts. This systematic removal of contextual information helps explain why algorithmic models often fail to work across different populations and settings—the situated knowledge being stripped away may contain information about important social and environmental factors that shape health outcomes differently across communities (c.f. Obermeyer et al. 2019; Seyyed‐Kalantari et al. 2021).

Decontextualisation is intensified when data is shared across research networks. Participants described the challenges of creating coherent datasets from information collected under different protocols and in different settings. As one researcher explained regarding imaging data:

If [the participant] moves in the scanner that has a big impact on the results. So how you account for that movement varies quite a lot. There’s a big problem that everyone does that pre‐processing in a slightly different way and therefore comes up with slightly different results. There was a study a couple of years ago, where they sent the same dataset to 10 different labs and said, what's the answer? And they got 10 different answers pretty much. Because of the different way that the same data set have been processed.

(Interviewee 10)

This example reveals the situated practices of data collection and processing—the specific ways different laboratories handle participant movement, their particular preprocessing protocols and their institutional norms—that must be erased to create the standardised formats that risk algorithms require. The acknowledgement that identical data can produce ‘10 different answers’ demonstrates the social construction of knowledge outlined earlier; what appears as objective analysis actually involves contextual interpretive work.

Participants noted that standardisation becomes increasingly challenging as new forms of data and computational techniques emerge:

Everyone is doing something different… for established processes you can have an SOP [standard operating procedure]. But when you're doing research stuff, sometimes you don't even know necessarily exactly what…you want to look at. But there's lots of different rabbit holes you can go down. So I think people are doing different things.

(Interviewee 5)

These accounts reveal how the drive toward standardisation encounters ongoing resistance from the heterogeneity of research practices and data collection contexts. Even as researchers work to create the decontextualised formats that algorithms require, new technologies and research approaches provide fresh forms of contextual variation that resist algorithmic standardisation.

The tension between situated knowledge and computational requirements becomes particularly acute when researchers work with clinical data not originally collected for research purposes. Participants described encountering data that contains clinically meaningful information, but lacks the precise specifications required for their algorithms:

Even for instance, the one I said for ischemic heart disease—for the computer, it wants to know not only the location, but the duration. But humans‐ when cardiologists look at the images, they just see ‘oh this patient had an attack here’, that's it. They will not write ‘okay the length of the heart attack is this, it's so long’, they don't find all this information on the images. They just write: this patient had a heart attack. All these are terms that we take for granted, but … the computer is a precise machine, its precision is important.

(Interviewee 4)

These examples illustrate how operationalising risk through algorithms requires the systematic removal of situatedness in practices of standardising, sharing and processing data. Data are not objectively collected and analysed, but undergoe multiple reconfigurations as it circulates through research networks. Each stage of processing, from initial cleaning to cross‐institutional sharing to algorithmic preparation, strips away the situated knowledge embedded in the original production contexts. The specific clinical encounters, institutional practices, local protocols and contextual judgements that shaped data collection must be erased to create the universal portable formats that risk algorithms require.

This reveals a fundamental tension between situated knowledge and algorithmic risk analysis: The more data is made compatible with computational processing, the more it is divorced from the situated contexts where health and disease actually unfold. However, this decontextualisation process also creates challenges in obtaining sufficient data to satisfy algorithmic requirements: by stripping away contextual detail to make data compatible with algorithms, researchers can leverage powerful analytic tools, but this very process also exposes the limits of available data, to which we now turn.

### 4.3 The Gap Between Data and Algorithms

Previous sections examined how the imaginary is enacted in practice; this section focuses on how it is sustained and reproduced over time. In particular, we show how operationalising risk through systematic decontextualization creates ongoing challenges in obtaining sufficient data to satisfy algorithmic requirements, leading to a persistent gap between algorithmic capabilities and data availability. Participants described a fundamental asymmetry in which algorithmic capabilities consistently outpace the data that can be generated or accessed.

Although algorithms can analyse increasingly complex relationships, they demand massive datasets that often exceed researchers' capacity to produce or obtain sufficient data. Beyond the challenges of sharing existing data already discussed, there are also practical limitations in data generation:

I think the challenge is getting enough patients recruited into clinical trials, or into the studies that we run. The harmonisation between different bits of data from different places as well is a challenge. So, if I do a study here, someone else does a study elsewhere, it'd be great to be able to combine those data so that we can have a bigger dataset. But there's different scanners, different ways of analysing the data, slightly different way, slightly different scans means it can be difficult to do that.

(Interviewee 3)

We argue that this gap between algorithmic capacity and data availability is not simply a technical limitation but an important sociomaterial space that is fundamental in generating ongoing engagement, investment and optimism for algorithmic research. In this sense it can be seen as a *productive* gap. Rather than undermining confidence in data‐driven approaches, we suggest the gap sustains the imaginary of data‐driven health insights via three key functions.

First, it opens up space for researchers to attribute any shortcomings of research to a lack of data rather than questioning algorithmic approaches:

The algorithm needs a large enough sample size to build a model that's reliable, that's the main thing. Now the algorithm is getting more and more advanced. Even the deep learning … depend on thousands, if not tens of thousands, learning samples… So I don't think there is a limitation on the algorithm side, for now it's already going quite ahead of what our data can capture. Our data will not catch up with the power of the algorithm.

(Interviewee 11)

Indeed, the perceived value of algorithmic approaches was actually enhanced in the context of data limitation, as algorithms demonstrated their capacity to extract meaningful insights from what would previously have been considered inadequate data:

It’s funny because when I started doing this, I had a very clear idea what the data should look like. And well it can't be accurate, if you're not doing it how we used to do it. But I have been absolutely amazed at what the deep learning has allowed us to extract from the data, despite it not looking quite as pretty. So I think it was like my preconceptions in a way have changed as technology analysis has improved. … we've developed methods that have overcome everything. … It has changed the way I think about things.

(Interviewee 20)

This illustrates the mutual transformation (Knorr‐Cetina 2005) of researchers and algorithms. As technological development has evolved over the years and algorithms have appeared in new configurations and achieved new capabilities, researchers' perspectives on what constitutes ‘adequate’ data and what can be accomplished with it have fundamentally shifted. In this way, each new algorithmic advance reframes previous limitations as temporary obstacles rather than fundamental constraints, thus sustaining the imaginary.

The second imaginary‐sustaining function of the gap is to create an imperative for ever‐expanding data collection, justified by the promise that sufficient volume will eventually bridge the divide between algorithmic potential and current limitations. This drives speculative accumulation of data, as this participant describes:

The data will keep growing exponentially or probably even more than exponentially. And simply because we are so active in this space… So, all this data needs to be stored, at least temporarily stored, and made sense of. I think for our advancement we need all the data. So it makes [sense to] … just store everything. And at some point, some can be deleted if it can be regenerated.

(Interviewee 1)

This imperative to ‘store everything’ means collecting data that might become useful at some point in the future. We refer to this as ‘rainy day’ data; data without immediate application but whose value is linked to anticipated future utility. However, if the rainy day ever arrives, hypothetical future analysts would inevitably confront the same processing challenges and data‐algorithm gaps that characterise current research. Thus, we can see data reuse itself as a sociotechnical imaginary—a compelling vision of future value extraction that shapes present practices despite no concrete evidence it will prove valuable. Here, the imperative to collect more data continues unabated, driven by the persistent belief that accumulation can solve the algorithmic‐data gap rather than questioning the fundamental approach.

This leads us to the third imaginary‐sustaining function of the data gap, which involves driving closer relationships between academia and industry. The quest for ever more data has reshaped health research infrastructure, creating demands for increasingly sophisticated systems for sharing, storing and accessing information. This has led to deepening entanglements between academic institutions and commercial technology providers who possess more advanced computational capabilities, as reflected by these participants:

I work with lots of companies with Biobank data and in a way, we have to work with companies because they have the resources that we don't, [for] computing generally.

(Interviewee 20)

These partnerships with commercial entities support algorithmic imaginaries by making their promises appear more achievable, whereas sustained industry investment and continuous innovation reinforce optimism about data‐driven research. Participants observed that major technology corporations can invest significantly more in developing computational resources than academic institutions ever could:

Cloud computing has a real edge, all these like Google Cloud or Amazon AWS, just because they spend so much money into optimising their data centre. They have entire teams of data scientists and engineers and it's just the kind of resources that smaller data centres just don't have… [universities] want the most powerful computers at the cheapest cost, that’s usually the deal, it’s like we want power but we don’t want to put too much money into it.

(Interviewee 12)

Participants observed that the integration of industry partnerships had become thoroughly embedded in the research ecosystem, with commercial collaborations now a fundamental component of developing and sustaining modern data infrastructure:

It's changed a lot in the last few years and it continues to change. So maybe 10 years ago, we always had to go and work at the hospital on their computers, physical computers, but more and more hospitals have set up virtual access. So in order to work on the records, I still have to log into the hospital computers but I could do that remotely, in this case from my home, but I'm actually still working within the hospital’s infrastructure if you like. But then some hospitals have started to shift the storage of their health records onto cloud services and there are a few now who have appropriate security models and governance structures in place to allow them to host their data on, say, Microsoft Azure or on Amazon's cloud.

(Interviewee 24)

This represents a significant shift from previous research systems, reflecting a fundamental transformation in how scientific infrastructure is conceived, funded and maintained in recent years. Beyond simply providing practical solutions, these partnerships serve as proof of concept that the algorithmic imaginary is achievable.

As we have shown, the three functions of the data‐capacity gap work together to configure a research landscape where the gap becomes not only a problem to be solved but also a productive organising principle that sustains the imaginary by driving the pursuit of ever‐increasing volumes of data. This creates a self‐reinforcing circular logic: Algorithmic approaches require more data to succeed; therefore, all possible data must be collected; yet this abundance creates processing challenges that necessitate more sophisticated algorithms, enhanced storage and greater computing resources, which in turn drive the collection of even more data. This pursuit appears to be Sisyphean—as algorithms evolve, so too does the threshold of ‘sufficient data’, fuelling an endless cycle of collection that can never be satisfied.

Despite evidence of their inability to fully process current datasets, the imperative to collect more data continues unabated, driven by the persistent belief that the algorithmic–data gap can be solved through further data accumulation. This circular logic reveals why situated approaches struggle to gain traction—any suggestion to work with smaller contextually rich datasets appears inadequate compared to the promise of algorithmic solutions operating on massive scales. These three mechanisms demonstrate how sociotechnical imaginaries are actively configured through everyday research practices rather than existing as abstract visions. In the following discussion, we examine how understanding this process of configuration might open possibilities for reshaping health data research.

## 5 Towards Situated Alternatives

Our analysis reveals how sociotechnical imaginaries about data‐intensive healthcare are sustained through three interconnected processes operating in everyday research practice. We have demonstrated how risk narratives provide the organising logic that brings diverse data assemblages into meaningful existence and justifies the collection and analysis of ever‐expanding datasets. Operationalising these narratives through algorithmic approaches requires translation processes that systematically strip away contextual information in order to render data computationally compatible. Decontextualisation is therefore not incidental but constitutive of algorithmic health research.

These decontextualisation practices are not just technical adjustments but are structured by the risk‐oriented logic that organises algorithmic medicine. As Amoore (2016) argues, algorithmic systems operate through anticipatory logics that privilege correlation and prediction over explanation. This shift is reflected in our findings, where researchers prioritised elucidating risk over aetiology. Within such configurations, situated forms of knowledge struggle not because they lack relevance but because they are structurally misaligned with systems organised around abstraction, scalability and predictive performance. Context appears as noise and variation becomes a technical problem to be resolved. In this way, epistemic exclusion is built into the configuration itself, as forms of expertise concerned with context, interpretation and lived experience seem irrelevant.

Yet decontextualisation also creates ongoing challenges in obtaining sufficient data to satisfy algorithmic requirements. The labour‐intensive processes of cleaning, standardising and harmonising data, combined with the volumes treated as necessary for algorithmic performance generate a persistent gap between algorithmic capacity and data availability. We conceptualise this as a productive gap. Rather than undermining confidence in data‐driven research, the gap sustains the imaginary by allowing limitations to be attributed to insufficient data and not the epistemic assumptions embedded in algorithmic approaches. The solution then appears to be more data, more harmonisation and more infrastructure. In this way, the gap stabilises the very imaginaries that generated it.

This gap between data availability and usability is evident at other stages of the data cycle, with similarly productive effects. In their study of practitioners responsible for producing and curating data, Bates et al. (2025) use the term data dilemma to describe the mismatch between the vast volumes of data available and the amount suitable for algorithmic training. They show how this paradox of abundance and insufficiency does not destabilise ambitions for algorithmic approaches but generates pressure on data producers to create more and ‘better’ datasets to meet demand. This resonates with a growing body of research on ‘data work,’ which foregrounds the everyday labour required to produce, stabilise and circulate data (Ribes and Jackson 2013; Bates et al. 2016). This literature emphasises that data are not neutral inputs plugged into algorithmic systems, but are constituted through situated practices of cleaning, formatting, translating and aligning them to particular infrastructural requirements (Bowker and Star 1999; Gitelman 2013). Bates et al. (2016) show that in the process of moving between sites, for example from clinic to database and from database to model, data are reshaped and transformed. Similarly, our interviews reveal the processes through which contextual meaning is systematically reconfigured in order to produce data that are mobile and compatible within computational systems.

Given the central role we have shown the data gap plays in sustaining imaginaries of data‐driven solutions, we suggest it marks an important site for intervention. As Suchman (2012) argues, configurations might be reconfigured at such points of stabilisation. If the productive gap continually renews commitments to scale, then this is where attempts to short‐circuit the cycle should be directed. This does not require abandoning large‐scale datasets or algorithmic approaches, but challenging the assumption that decontextualisation is necessary for algorithmic analysis. The question is how to value context within data practices, rather than continually removing it.

Gabrys et al.’s (2016) work on ‘just good enough data’ in environmental sensing offers one example of how alternative configurations might work. Challenging the convention that sensor data must be standardised to be valid, they composed sensor readings into ‘data stories’ that integrate measurements with residents' situated knowledge, such as when and where to monitor, what patterns seem unusual, and how readings relate to local activity. In this way, context becomes the analytic material that gives measurements meaning rather than noise requiring elimination. Such approaches suggest that health data research need not choose between scale and situatedness; the issue is not data volume but the assumption that only decontextualised algorithm‐ready data constitute legitimate evidence.

Such examples demonstrate that alternative configurations are possible. However, translating these principles into health data research, where infrastructures, algorithmic pipelines and institutional arrangements are already deeply established, presents distinct challenges. Creating space for situated approaches therefore requires methods that make these existing configurations empirically visible. Several methodological approaches offer starting points. Bates et al.'s (2016) data journeys approach follows data across sites of practice, revealing where contextual meaning is reformatted to meet computational demands. Tkacz et al.'s (2021) data diary method documents data as situated practice, tracing production and transformation within specific contexts while facilitating collaboration. Abildgaard's (2025) ‘sensing data’ methodology incorporates researchers' positionality, making explicit how embodied perspectives shape interpretation. Together, these approaches treat context as constitutive of data rather than extraneous information to be eliminated. Making configurations visible in this way opens what Haraway (2016) terms ‘other worldings’: possibilities for organising research that do not yet exist within current arrangements.

This paper traced how sociotechnical imaginaries are enacted in health data research to identify junctures where reconfiguration may be possible. By revealing three interconnected processes that stabilise these imaginaries, we showed how a productive gap generated by decontextualisation practices drives ongoing data accumulation. We argue that this productive gap represents the critical juncture for intervention. Rather than continually justifying more data collection, it reveals where assumptions about decontextualisation might be challenged. Creating space for situated knowledge in data‐intensive health research requires collaborative approaches that accommodate epistemic practices that value context over abstraction.

## Author Contributions

**Kate Lyle:** conceptualization, formal analysis, writing – original draft, writing – review and editing. **Gabrielle Samuel:** methodology, data curation, writing – review and editing, funding acquisition, conceptualization. **Anneke Lucassen:** funding acquisition, writing – review and editing.

## Funding

This study was funded by Wellcome, Grant Nos. 208053/Z/17/Z and 222180/Z/20/Z and MRC Grant No. MR/X021351/1.

## Ethics Statement

This study received ethical approval from King's College Research Ethics Committee (MRM‐21/22‐26574).

## Consent

Informed consent was obtained from all individual participants included in the study.

## Conflicts of Interest

The authors declare no conflicts of interest.

## Data Availability

The data supporting this study are not publicly available because of privacy or ethical restrictions but are available from the corresponding author upon reasonable request, and on the basis of the consent that was provided by participants.
